# A Case of Obstructive Jaundice due to Bile Duct Tumor Thrombus of Hepatocellular Carcinoma Diagnosed by Peroral Cholangioscopy

**DOI:** 10.1002/deo2.70228

**Published:** 2025-10-22

**Authors:** Keisuke Kinoshita, Mizuki Endo, Tomoko Tokumaru, Tomoko Saito, Takuro Uchida, Masao Iwao, Mie Arakawa, Kazuhisa Okamoto, Masaaki Kodama, Kazunari Murakami

**Affiliations:** ^1^ Department of Gastroenterology Faculty of Medicine Oita University Oita Japan

**Keywords:** bile duct tumor thrombus, endoscopic retrograde cholangiopancreatography, hepatocellular carcinoma, obstructive jaundice, peroral cholangioscopy

## Abstract

While hepatocellular carcinoma (HCC) often invades the portal or hepatic vein to form tumor thrombus, tumor thrombus in the bile duct is rare. In such cases, differentiation from intrahepatic cholangiocarcinoma is difficult, and the tumor often appears as a smooth, yellowish‐white, polypoid mass within the bile duct lumen. We report herein a case of obstructive jaundice due to bile duct tumor thrombus of HCC diagnosed by peroral cholangioscopy (POCS). A 64‐year‐old man presented with epigastralgia and jaundice. Contrast‐enhanced computed tomography revealed an irregular mass with hypoenhancement in liver segment S8, along with dilatation of the right intrahepatic bile duct due to the invading tumor. The hepatic mass was poorly visualized on ultrasound, making percutaneous liver tumor biopsy difficult. POCS was performed after endoscopic retrograde cholangiopancreatography for biopsy of the intrahepatic bile duct tumor thrombus. POCS clearly revealed a smooth, yellowish‐white, polypoid tumor in the right intrahepatic bile duct, and a biopsy of the tumor was performed under POCS. Based on the pathological findings, HCC was diagnosed, and chemotherapy with atezolizumab and bevacizumab was initiated.

## 1 Introduction

Hepatocellular carcinoma (HCC) often invades the portal or hepatic vein to form tumor thrombus, but tumor thrombus in the bile duct is rare. In such cases, differentiating the tumor thrombus from intrahepatic cholangiocarcinoma is difficult, but tumor thrombus of HCC often appears as a smooth, yellowish‐white, polypoid mass within the bile duct lumen during peroral cholangioscopy (POCS) (SpyGlass DS; Boston Scientific Corp., Marlborough, Massachusetts, USA) [1, 2]. In general, moderately differentiated HCC shows early staining on contrast‐enhanced computed tomography (CECT) with washout in the venous phase. However, some poorly differentiated HCCs do not show early staining, making preoperative differentiation from other malignancies difficult in some cases. Here, we report a case of obstructive jaundice due to bile duct tumor thrombus of HCC, in which biopsy of the bile duct tumor was performed under POCS using SpyBite MAX biopsy forceps. The pathological diagnosis was then determined to be HCC with intrahepatic bile duct tumor thrombus instead of intrahepatic cholangiocarcinoma.

## 2 Case Report

**FIGURE 1:**
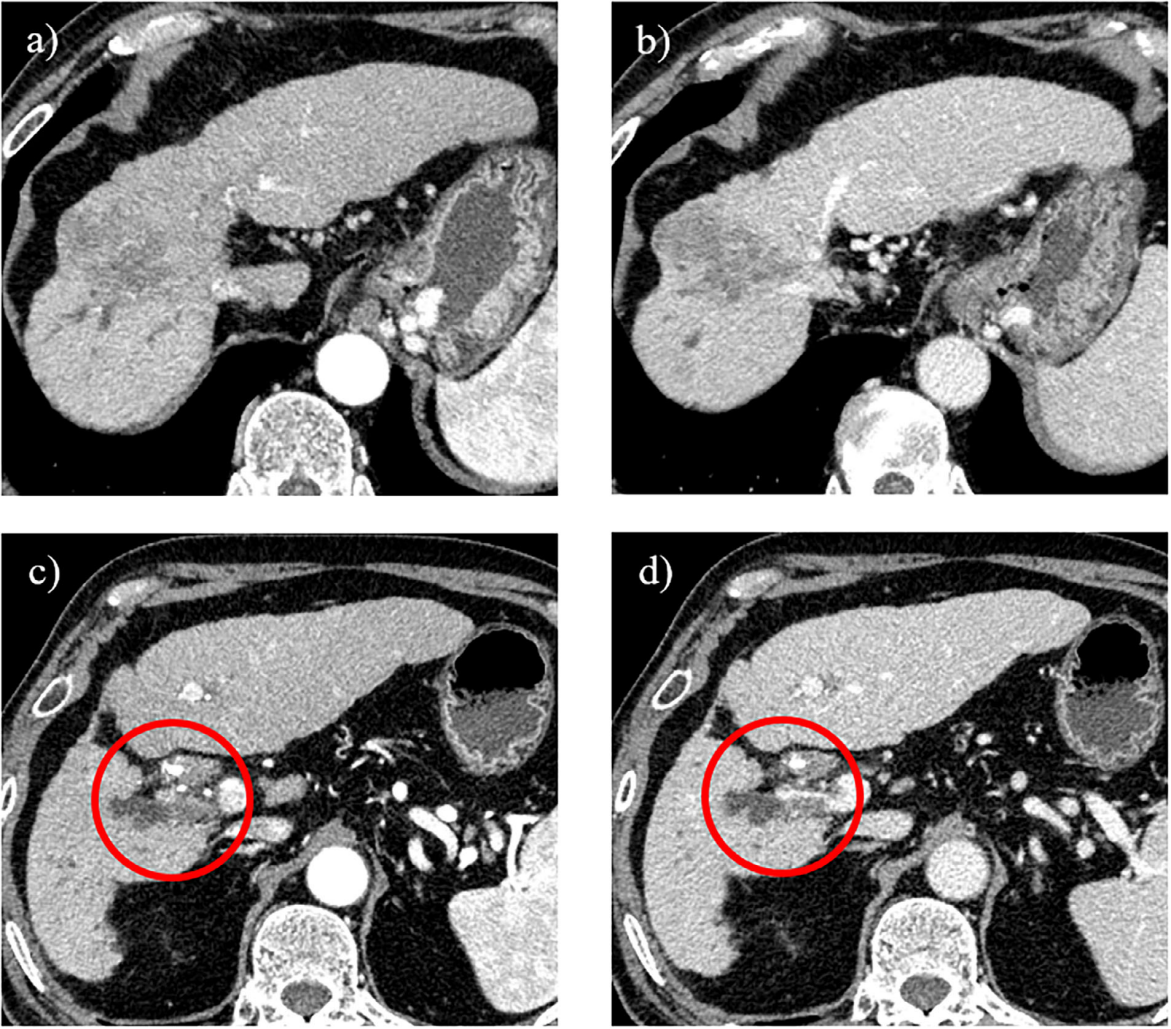
Findings on contrast‐enhanced computed tomography (CECT). (a) Arterial phase. On CECT, an irregular tumor without early staining is observed in liver segment S8 in the arterial phase. (b) Portal venous phase. On CECT, an irregular tumor with hypoenhancement and no washout is observed in liver segment S8 in the portal venous phase. (c) Arterial phase. CECT reveals dilatation of the right intrahepatic bile duct due to tumor invasion (red circle). (d) Portal venous phase. CECT reveals bile duct and portal vein invasion of the tumor and dilatation of the right intrahepatic bile duct due to tumor invasion (red circle).

**FIGURE 2:**
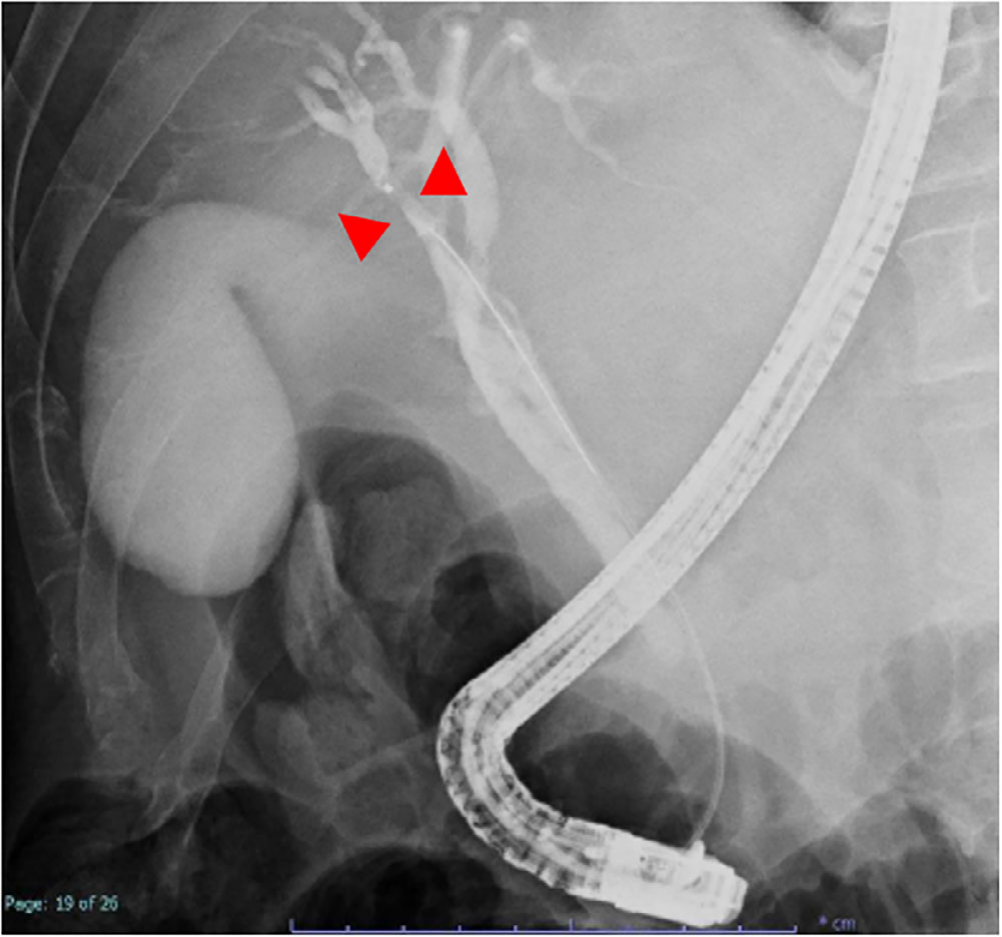
Findings on endoscopic retrograde cholangiopancreatography (ERCP). ERCP reveals stenosis of the right intrahepatic bile duct due to invade the hepatic tumor (red arrowhead).

**FIGURE 3:**
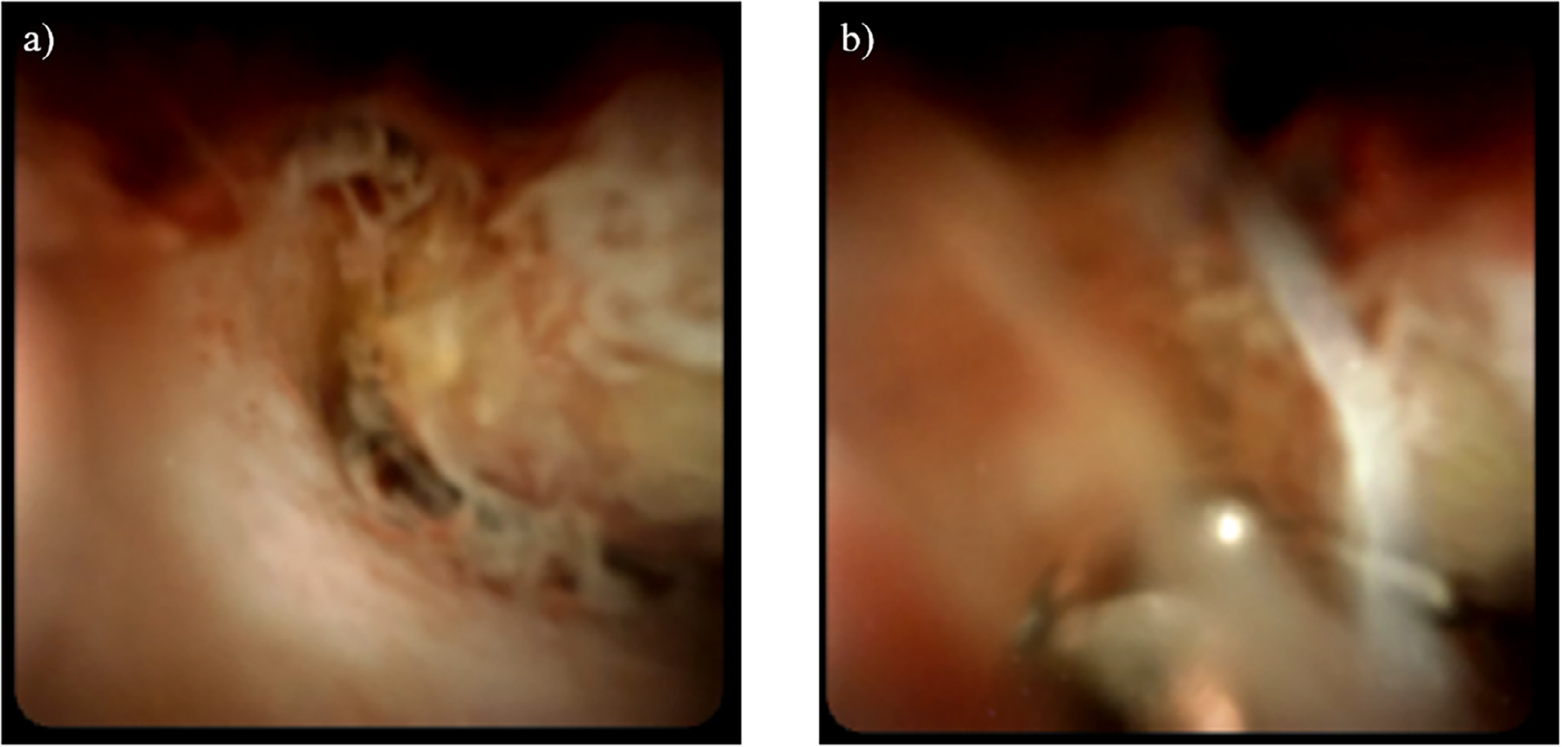
Findings on peroral cholangioscopy (POCS). (a) POCS clearly reveals a smooth, yellowish‐white, polypoid tumor in the right intrahepatic bile duct. (b) Biopsies from the tumor were performed three times under POCS using SpyBite MAX biopsy forceps.

**FIGURE 4:**
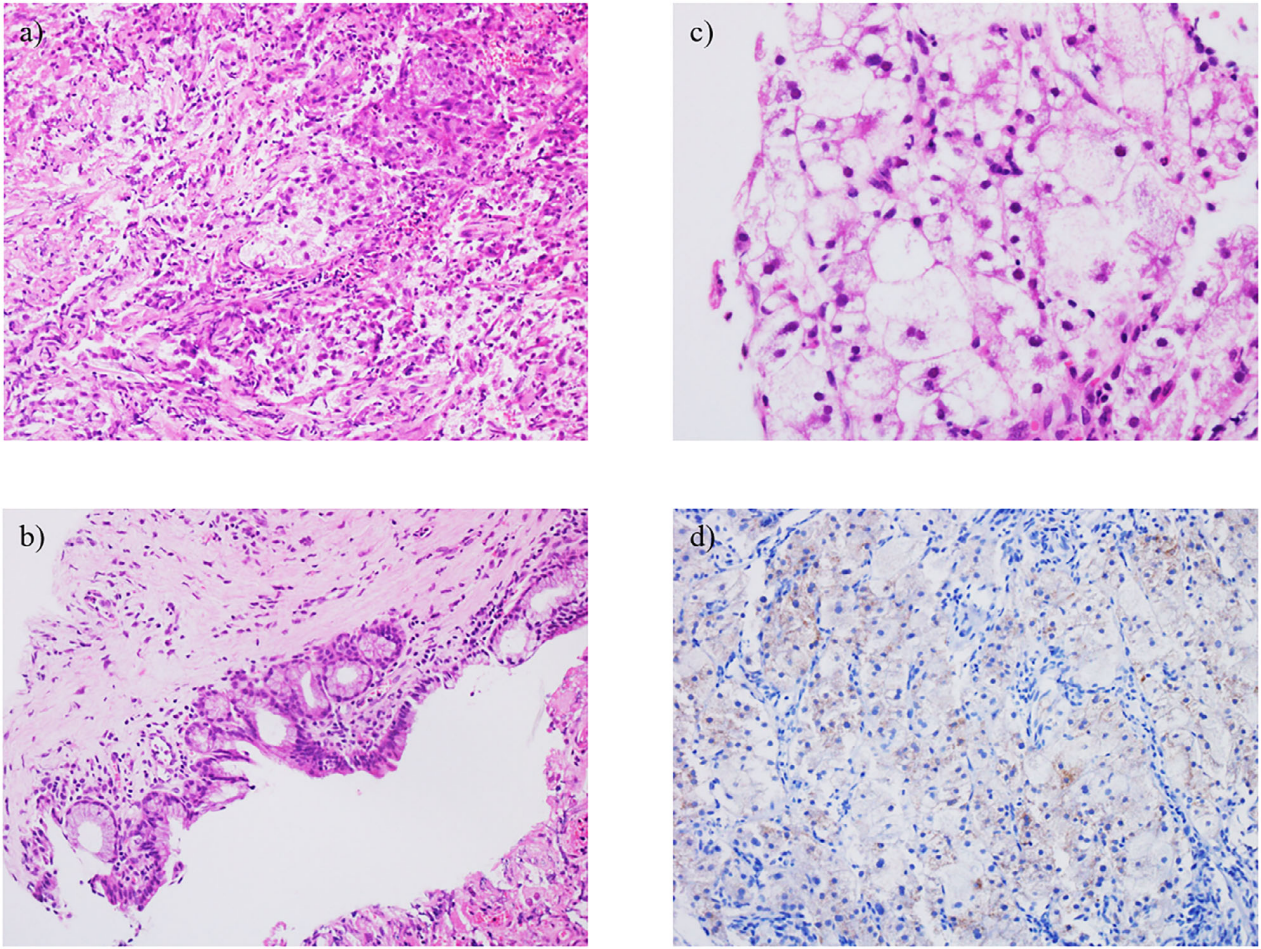
Pathological findings for the bile duct tumor thrombus. (a) Hematoxylin and eosin (HE) staining reveals the tumor as a cluster of poorly atypical cells with abundant foam cell‐like cytoplasm within fibrous stroma (200× magnification). (b) HE staining reveals tumor cells showing foam cell‐like cytoplasm (400× magnification). (c) HE staining also reveals normal bile duct epithelial cells with no atypical cells (200× magnification). (d) Tumor cells show positive Hep‐Per1 staining, leading to the diagnosis of hepatocellular carcinoma (HCC) (200× magnification).

A 64‐year‐old man had been treated with sofosbuvir and ribavirin for hepatitis C virus‐related cirrhosis at our hospital, achieving sustained virologic response (SVR), but had subsequently stopped attending regular appointments for 5 years. He presented with epigastralgia and severe jaundice (total bilirubin, 10 mg/dL; reference range, 0.4–1.5 mg/dL) and was referred to our hospital after CECT showed an irregular tumor with hypoenhancement in liver segment S8, along with dilatation of the right intrahepatic bile duct. At the time of referral to our hospital, the total bilirubin level had fallen to 1.65 mg/dL because a biliary stent had been placed in the common bile duct following endoscopic retrograde cholangiopancreatography (ERCP) in the previous hospital. Laboratory data revealed tumor markers of: alpha fetoprotein, 4.4 ng/ml; carcinoembryonic antigen, 2.8 ng/ml; carbohydrate antigen 19‐9 (CA19‐9), 87.9 U/ml; and protein induced by vitamin K absence or antagonist II (PIVKA‐II), 2036mAU/µl. Levels of CA19‐9 and PIVKA‐II were elevated. CECT performed at our hospital also revealed an irregular tumor with hypoenhancement in liver segment S8, along with dilatation of the right intrahepatic bile duct due to tumor invasion (Figure 1). The hepatic tumor was poorly detected on ultrasonography, making percutaneous liver tumor biopsy difficult. As a hepatic tumor was suspected to be invading the right intrahepatic bile duct on CECT, ERCP was performed for planning POCS, and primarily for defining the pathological diagnosis. ERCP revealed stenosis of the right intrahepatic bile duct due to invade the hepatic tumor (Figure 2). Based on these ERCP findings, it was considered that the liver tumor was exposed within the right intrahepatic bile duct, so POCS was performed for biopsy of the tumor thrombus in the right intrahepatic bile duct. POCS clearly revealed a smooth, yellowish‐white, polypoid tumor in the right intrahepatic bile duct, and biopsies from the tumor were performed three times under POCS using SpyBite MAX biopsy forceps (Figure 3 and Video S1). No complications were observed following biopsies from the tumor were performed three times under POCS using SpyBite MAX biopsy forceps. Pathological findings revealed clusters of poorly atypical cells with abundant foam cell‐like cytoplasm within the fibrous stroma upon hematoxylin and eosin staining, and positive Hep‐Per1 staining was a definitive finding (Figure 4). Based on these results, we diagnosed HCC. As normal bile duct epithelium was also present in the biopsy specimen, the diagnosis of intrahepatic cholangiocarcinoma was ruled out. The patient was diagnosed with intrahepatic bile duct tumor thrombus of HCC rather than intrahepatic cholangiocarcinoma, and was not indicated for surgery due to poor liver function, and was able to be treated with atezolizumab and bevacizumab chemotherapy.

## 3 Discussion

HCC is a disease with poor prognosis, resulting in the third highest number of deaths among malignant neoplasms worldwide and the fifth in Japan [3]. Conventional HCC is considered to be associated with chronic liver inflammation and cirrhosis caused by hepatitis B or C virus infection, alcoholic liver disease, or non‐alcoholic steatohepatitis. Recent advances in viral therapy using nucleic acid analogs against the hepatitis B virus (HBV) and direct‐acting antiviral (DAA) agents against the hepatitis C virus (HCV) have reduced the number of HCC cases caused by HBV or HCV. The high SVR rate among patients receiving DAAs means the cumulative carcinogenesis rate is higher than during the interferon therapy era [4, 5]. Indeed, the present patient also achieved SVR with sofosbuvir‐ribavirin treatment for HCV‐related cirrhosis, but the case involved highly fibrotic, non‐compensated cirrhosis. We therefore considered that this case also required careful follow‐up every 3–6 months.

HCC often invades the portal or hepatic vein to form tumor thrombus, but tumor thrombus in the bile duct is rare. In such cases, differentiation from intrahepatic cholangiocarcinoma is difficult, and the tumor often forms a smooth, yellowish‐white polypoid mass within the bile duct lumen during POCS [1, 2]. HCC that causes obstructive jaundice due to tumor thrombus in the bile duct is called icteric‐type hepatoma [6]. According to the 23rd National Follow‐up Survey of Primary Liver Cancer in Japan, HCC causing obstructive jaundice due to tumor thrombus in the bile ducts comprises 4.1% of all liver cancer cases [7]. HCC causing obstructive jaundice due to tumor thrombus in the bile ducts is often quite advanced at the time of onset, and hepatic resection has been considered difficult. Further, HCC causing obstructive jaundice due to tumor thrombus in the bile ducts is often difficult to differentiate from intrahepatic cholangiocarcinoma preoperatively. In general, well‐differentiated HCC commonly exhibits isovascular patterns in both arterial and delayed phases, whereas moderately differentiated HCC stains early in the arterial phase on CECT, with washout in the venous phase [8]. However, some poorly differentiated HCCs do not show early staining, making preoperative differentiation from other malignancies difficult in some cases. Similarly, in this case, the hepatic tumor, which led to the consideration of poorly differentiated HCC due to the absence of early staining, was also atypical and difficult to diagnose because CECT did not reveal early staining in the arterial phase or washout in the venous phase, and an irregular tumor with hypoenhancement was observed in liver segment S8, along with dilatation of the right intrahepatic bile duct due to the tumor invasion. In addition, the hepatic tumor was poorly detected by ultrasound, making percutaneous liver tumor biopsy difficult. As the hepatic tumor was suspected to be invading the right intrahepatic bile duct on CECT, we performed ERCP for planning POCS, and primarily for defining the pathological diagnosis. ERCP suggested stenosis of the right intrahepatic bile duct due to the hepatic tumor. Based on these ERCP findings, it was considered that the liver tumor was exposed within the right intrahepatic bile duct, so POCS was performed for biopsy of the tumor thrombus from the right intrahepatic bile duct. POCS clearly revealed a smooth, yellowish‐white, polypoid tumor in the right intrahepatic bile duct. Biopsy of the tumor was performed under POCS. This case thus demonstrated that POCS can be used to diagnose HCC with bile duct invasion, enabling direct morphological assessment, acquisition of reliable tissue sampling, and direct visualization‐guided targeted biopsy from the tumor, thereby facilitating differentiation from intrahepatic cholangiocarcinoma. To the best of our knowledge, no reports have provided detailed direct endoscopic observation of bile duct thrombus in HCC. We believe that we were able to capture the diverse pathophysiology of bile duct tumor thrombus in HCC.

In conclusion, HCC with obstructive jaundice due to tumor thrombus in the bile ducts is very rare. In this case, POCS proved useful in allowing direct observation of the HCC invading the bile duct, reliable biopsy under direct visualization, and pathological diagnosis of HCC with tumor thrombus in the intrahepatic bile duct, rather than intrahepatic cholangiocarcinoma.

## Author Contributions

**Conceptualization**: Keisuke Kinoshita; **Formal analysis and investigation**: Keisuke Kinoshita and Kazuhisa Okamoto; **Writing—original draft preparation**: Keisuke Kinoshita; **Writing—review and editing**: Mizuki Endo, Tomoko Tokumaru, Tomoko Saito, Takuro Uchida, Masao Iwao, Mie Arakawa, Kazuhisa Okamoto, Masaaki Kodama, and Kazunari Murakami; **Supervision**: Kazunari Murakami. All authors have approved the final version of the paper.

## Conflicts of Interest

The authors declare no conflicts of interest.

## Supporting information




**Findings on peroral cholangioscopy**. Peroral cholangioscopy clearly revealed a smooth, yellowish‐white, polypoid tumor within the right intrahepatic bile duct lumen, and a biopsy from the tumor was performed under peroral cholangioscopy. Intrahepatic bile duct epithelium was normal.

## Data Availability

The data that support the findings of this study are available from the corresponding author upon reasonable request.
